# Urothelial cancer proteomics provides both prognostic and functional information

**DOI:** 10.1038/s41598-017-15920-6

**Published:** 2017-11-17

**Authors:** Guillermo de Velasco, Lucia Trilla-Fuertes, Angelo Gamez-Pozo, Maria Urbanowicz, Gustavo Ruiz-Ares, Juan M. Sepúlveda, Guillermo Prado-Vazquez, Jorge M. Arevalillo, Andrea Zapater-Moros, Hilario Navarro, Rocio Lopez-Vacas, Ray Manneh, Irene Otero, Felipe Villacampa, Jesus M. Paramio, Juan Angel Fresno Vara, Daniel Castellano

**Affiliations:** 1 Department of Medical Oncology, University Hospital 12 de Octubre, i + 12, Madrid, Spain; 2 Molecular Oncology & Pathology Lab, INGEMM, Instituto de Investigación Hospital La Paz-IdiPAZ, Madrid, Spain; 3 Biomedica Molecular Medicine, Madrid, Spain; 4 Department of Pathology, University Hospital 12 de Octubre, Madrid, Spain; 5 Department of Statistics, Operational Research and Numerical Analysis, University Nacional Educacion a Distancia (UNED), Madrid, Spain; 6 Department of Urology, University Hospital 12 de Octubre, Madrid, Spain; 7 Molecular and Cell Oncology Group, Biomedical research Institute, University Hospital 12 de Octubre, i + 12, and Molecular Oncology Unit, CIEMAT, Madrid, Spain; 8 CIBERONC, Madrid, Spain

## Abstract

Traditionally, bladder cancer has been classified based on histology features. Recently, some works have proposed a molecular classification of invasive bladder tumors. To determine whether proteomics can define molecular subtypes of  muscle invasive urothelial cancer (MIUC) and allow evaluating the status of biological processes and its clinical value. 58 MIUC patients who underwent curative surgical resection at our institution between 2006 and 2012 were included. Proteome was evaluated by high-throughput proteomics in routinely archive FFPE tumor tissue. New molecular subgroups were defined. Functional structure and individual proteins prognostic value were evaluated and correlated with clinicopathologic parameters. 1,453 proteins were quantified, leading to two MIUC molecular subgroups. A protein-based functional structure was defined, including several nodes with specific biological activity. The functional structure showed differences between subtypes in metabolism, focal adhesion, RNA and splicing nodes. Focal adhesion node has prognostic value in the whole population. A 6-protein prognostic signature, associated with higher risk of relapse (5 year DFS 70% versus 20%) was defined. Additionally, we identified two MIUC subtypes groups. Prognostic information provided by pathologic characteristics is not enough to understand MIUC behavior. Proteomics analysis may enhance our understanding of prognostic and classification. These findings can lead to improving diagnosis and treatment selection in these patients.

## Introduction

Urothelial cancer (UC) is responsible for approximately 165,000 deaths per year worldwide (GLOBOCAN 2012)^1^. Pathological classification divides UC into two major subtypes according to the invasion depth: non-muscle invasive and muscle invasive urothelial carcinoma (MIUC) but not molecular categorization is clinically indicated. However, the outcome and prognosis may be different across subsets of patients within same staging.

MIUC is characterized by a high risk of relapse and metastasise. Despite radical cystectomy with neoadjuvant cisplatin-based chemotherapy, the current risk of recurrence as well as mortality is nearly 50%^2^. In the adjuvant setting, chemotherapy is also associated with improved survival in patients with locally advanced bladder cancer^3^.

Pathological prognostic factors such as lymphovascular invasion, grade or molecular alterations are not currently modifying treatment choice. Large collaborative efforts have provided a more comprehensive view of the genomic landscape of MIUC identifying molecular subtypes that have yet to prove predictive value^3–5^. At present, no molecularly targeted drugs are approved for UC.

Before the genomic era, p53 was thought to be prognostic and predictive marker measured by immunohistochemistry in UC^6^. Several methodological issues questioned conflicting results including proteomics assessment^7^. In the last years, proteomics approaches have been incorporated into the study of clinical samples, as a way to complement the information provided by classical factors and genomics. Mass spectrometry-based proteomics have emerged as preferred components of a strategy for discovering diagnostic and prognostic protein biomarkers and as well as new therapeutic targets^8^. These investigations are very encouraging^9,10^ and the potential of tumor biomarkers discovery is unclear^11^.

Genomics advance in UC has not been translated into molecularly-based biomarker for treatment selection. Since few data is available with proteomics, we aimed to identify whether differentially expressed protein biomarkers in tumor tissue may predict different outcomes.

## Results

### Study Population

Fifty eight patients with a median age of 68 years (range 45–78 years) were included. Main characteristics are displayed in Table 1. After a median follow up of 38 months, 34 (58.6%) patients relapsed and 35 (60.4%) had died. Median follow-up of all patients was 34 months (range 3–114 months). Median distant disease free survival was 27.7 (27.2–45.1, 95%CI). Five- years-distant relapse free survival was: 75% in stage I/II, 45% in stage III and 25% in stage IV.

**Table 1:** Study population.

Urothelial tumors	
**Number of patients**	58
**Age (years)**	
≤60	**20(34,5%)**
>60	**38(65,5%)**
Median(IQR)	68(60–71)
Range	45–78
**Sex**	
Male	51(88%)
Female	7(12%)
**pT category**	
pT2a	2(3.5%)
pT2b	10(17.3%)
pT3a	27(46.5%)
pT3b	8(13.8%)
pT4a	9(15.5%)
pT4b	1(1.7%)
Missing	1(1.7%)
**pN category**	
pN0	32(55%)
pN1	14(24%)
pN2	6(10%)
Missing	6(10%)
**Highest G grade**	
G1-2	8(14%)
G3	44(76%)

### Protein preparation and mass spectrometry analysis

After mass spectrometry (MS) workflow, 58 urothelial tumors were analyzed. Raw data normalization was performed as described previously^12^. 4,405 protein groups were identified using Andromeda, of which 1,453 presented at least two unique peptides and detectable expression in at least 75% of the samples. No decoy protein passed through these additional filters.

### Protein expression analyses of urothelial tumors and identification of new molecular subtypes

**Figure 1:**
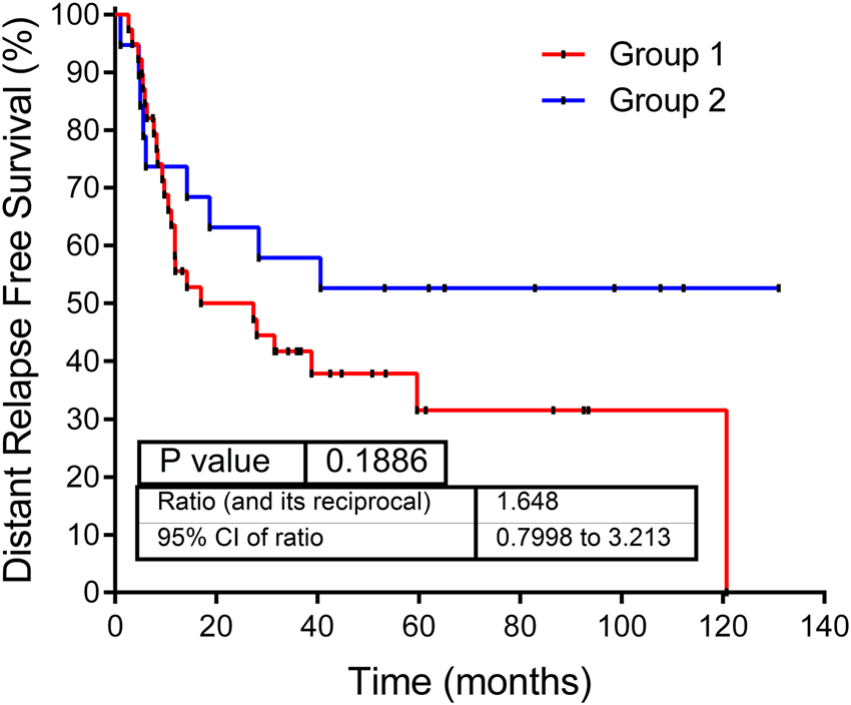
Kaplan–Meier survival curves obtained from high/low risk groups originated in our classification.

Proteomics data from 58 MIUC tumors were analyzed using sparse k-means and random-forest in order to establish a consistent classification of our samples. Using these approaches, two different molecular groups were identified on the basis of 34 proteins differentially expressed between both groups (Supplementary Figure 1, Supplementary Table 1). From those, 20 proteins have higher expression in group 1, including EHD2, FLNA and TNS1. Gene ontology analyses showed that these proteins are mainly related with focal adhesion and extracellular matrix. On the other hand, 14 proteins have higher expression in group 2, including HSBP1. Gene ontology analyses showed that these proteins are mainly related with transcription processes and immune response. Group 1 showed better prognosis than Group 2, although these differences were not significant (Fig. 1). Contingency analyses showed that these two groups are independent of clinical factors such as stage, tumor size and lymph node status.

### Network construction and functional node assignation

**Figure 2:**
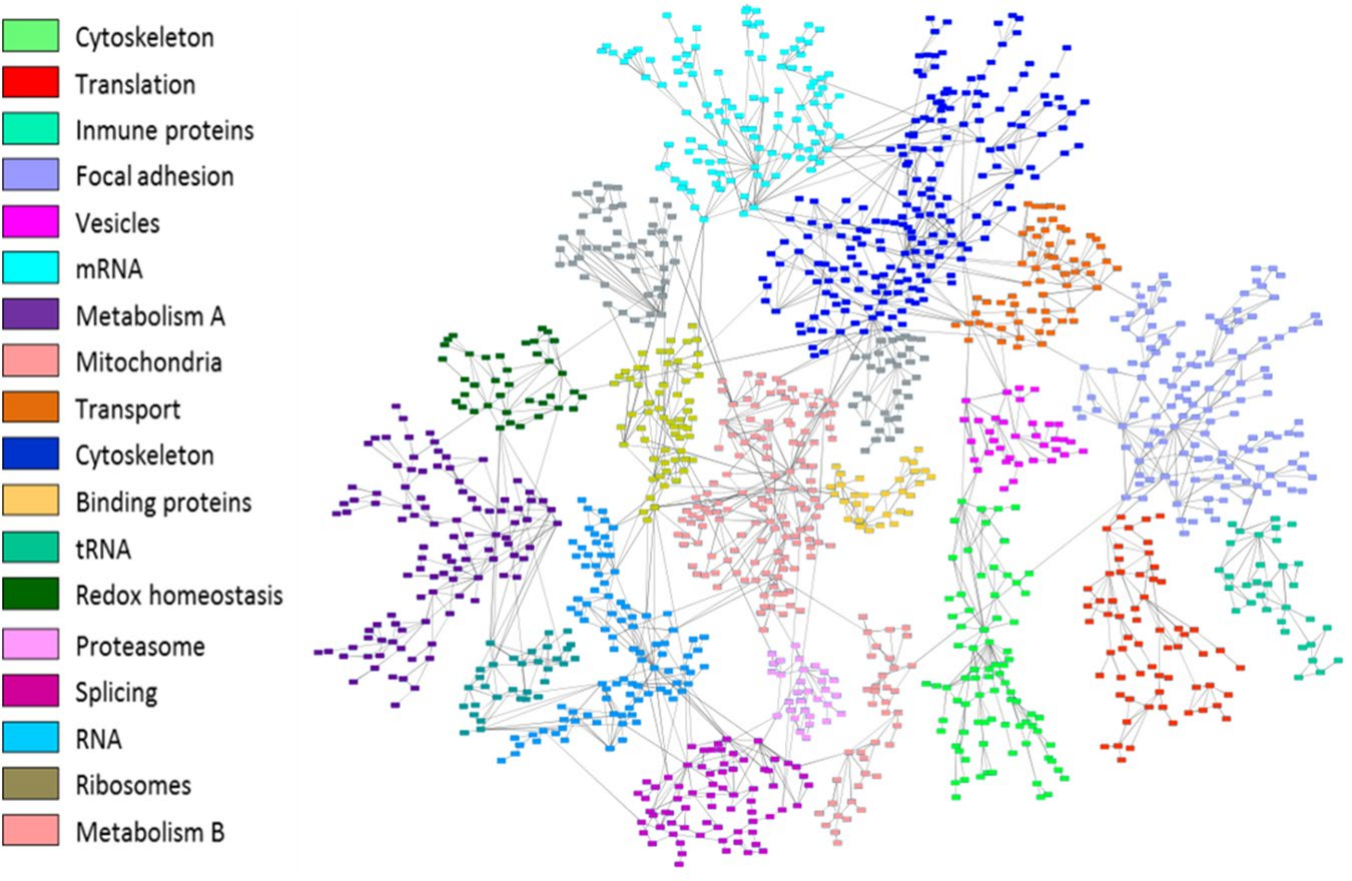
Probabilistic graphical model analysis unravels the functional organization of proteins in MIUC based on correlation. Grey nodes are nodes without any majority function assigned.

**Figure 3:**
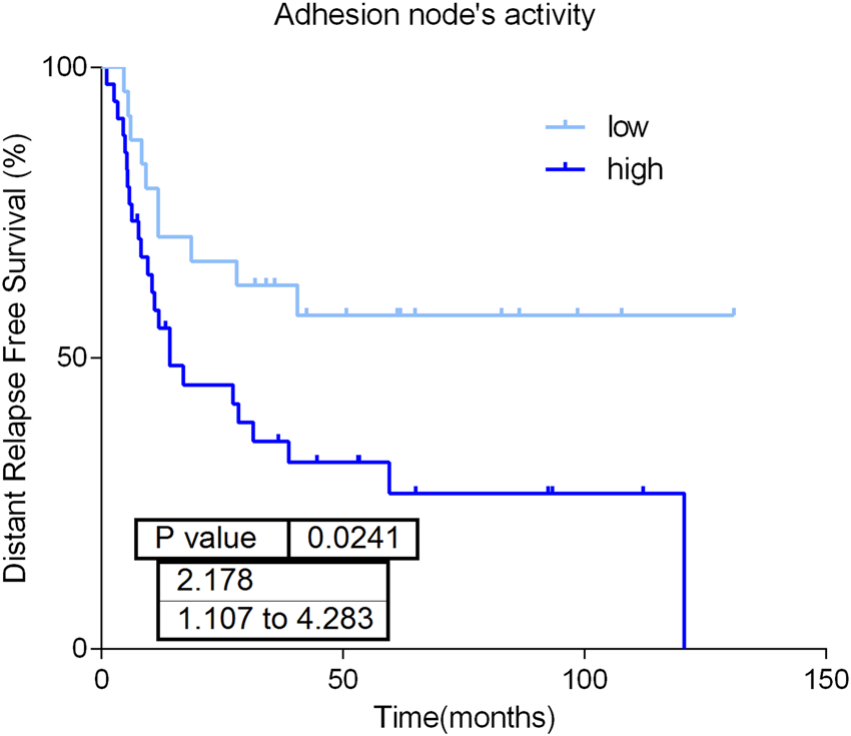
Focal adhesion node’s activity has prognostic value (p-value = 0.0241, HR = 2.178, IC95 = 1.107 to 4.283).

Protein expression data from all samples were used in the probabilistic graphical models analyses, with no other *a priori* information. The resulting graph was processed (Fig. 2) looking for a functional structure, i.e., if the proteins included in each branch of the tree had some relationship regarding their function, as previously described^12^. Thus, we divided our graph into eighteen branches, and performed gene ontology analyses. The structure of the probabilistic graphical model had a strong biological function basis. The next step was to calculate the activity for each branch with a specific biological function, i.,e., a functional node, as previously described^12^ (Supplementary Figure 2). Once calculated, we evaluated the prognostic value of each functional node activity in MIUC. Focal adhesion functional node activity splits the population into two groups with different prognosis (p = 0.0241, HR = 0.44 IC95 = 0.234 to 0.899) (Fig. 3). Afterwards, we assessed the differences in the functional nodes activities between Group 1 and Group 2 using class comparison analyses. Twelve nodes showed significant different activity between both groups. Focal adhesion, two cytoskeleton nodes, tRNA, ribosomes and metabolism A & B functional nodes showed increased activity in Group 1 tumors, whereas vesicles, transport, proteasome, RNA and splicing nodes showed increased activity in Group 2 tumors (Supplementary Figure 2).

### Focal adhesion functional node

Focal adhesion functional node includes twenty six proteins related with extracellular matrix and focal adhesion. COL1A1, SOD3, COL6A1, COL6A2, CAPN2, MSN, STOM, PRELP, NID2, DAG1, LPP and GPI are highly expressed in group 1 while SFN and HDLBP are highly expressed in group 2 (p < 0.05). Overall, functional activity of this node is higher in group 1. In addition, this functional node has prognostic value in our cohort.

### Development of a prognostic protein signature in MIUC

**Figure 4:**
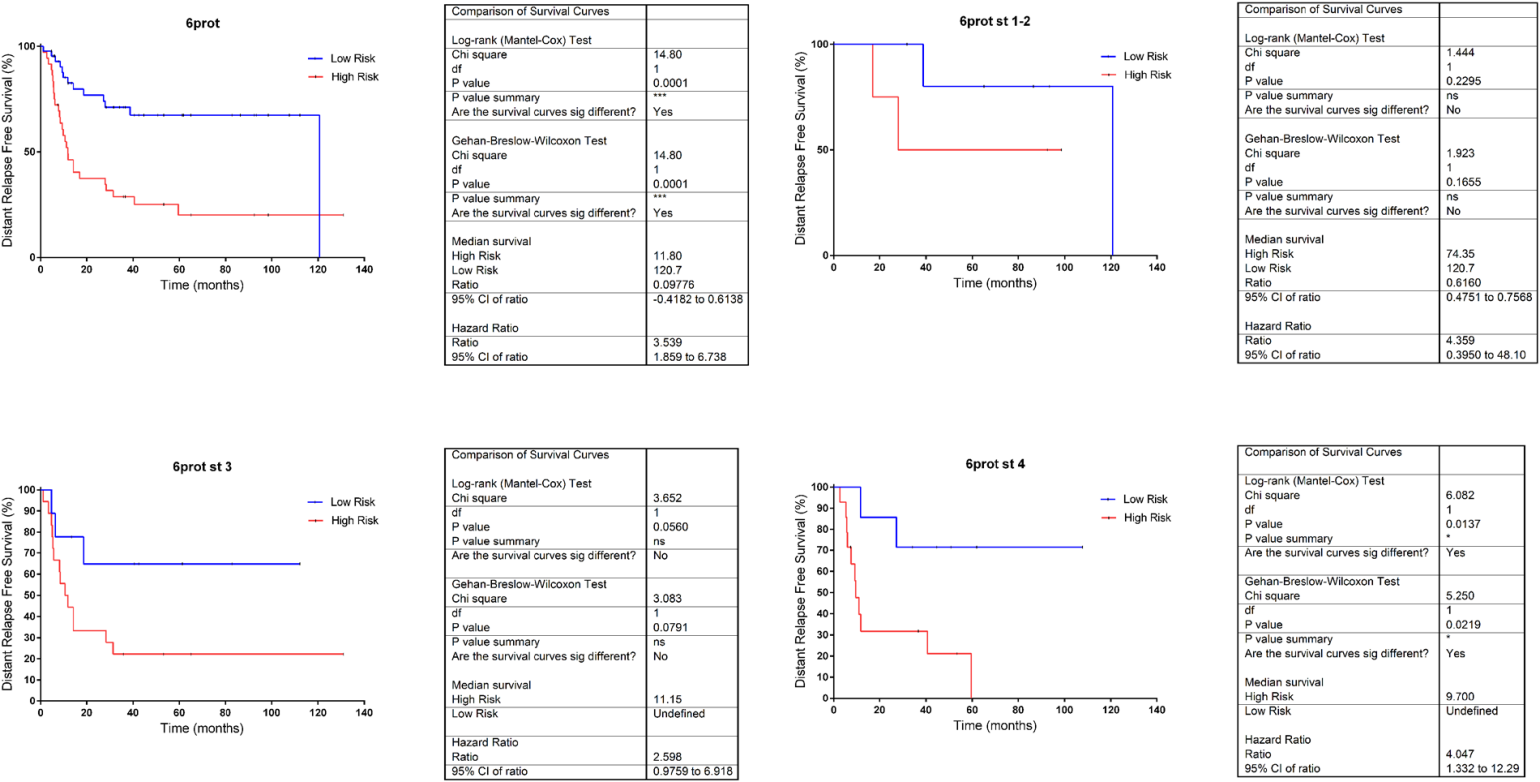
Prognostic signature composed by 6 proteins. A. All data. B. Stage 1–2. C. Stage 3 D. Stage 4.

66 proteins were found to be associated with recurrence risk in MIUC (Supplementary Table 2). A recurrence signature was developed as previously described^13^. Six proteins of these 66 were included in the prognostic signature: ANXA1 (Annexin A1), BGN (Biglycan), IGFBP7 (Insulin Like Growth Factor Binding Protein 7), ISLR (Immunoglobulin Superfamily Containing Leucine-Rich Repeat), MDP1 (Magnesium-Dependent Phosphatase 1) and PLS3 (Plastin 3). The recurrence protein score split our population into two risk groups with different five year distant relapse free survival: 70% vs. 20% (HR 3.53 95% CI 1.8–6.7; [p < 0.001]) (Fig. 4). The association between the score and DRFS was similar for patients with stage III and those with stage IV (Fig. 4). These results were verified using gene expression data from the MD Anderson cohort^4^. In this population, the 6 proteins predictor identifies two populations with different relapse risk (HR 2.10 95% CI 0.2–1.1; [p = 0.04]) (Supplementary Figure 3).

### Functional proteomics add prognostic information to prognostic signature

**Figure 5:**
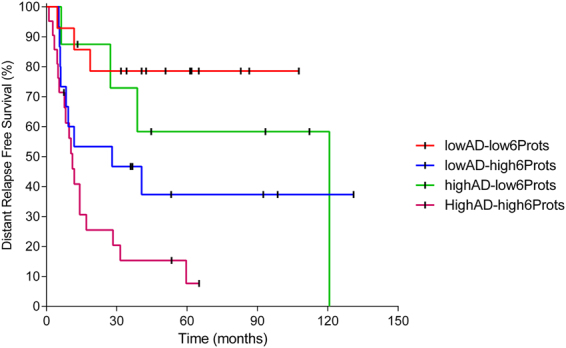
Kaplan–Meier curve curves showing overall survival based on 6 protein signature merged with focal adhesion node activity signature (p-value = 0.0003).

Information provided by the prognostic signature is complementary to the prognostic information provided by focal adhesion functional node activity signature and both predictors combined establish four different classes into the population with different relapse risk (p-value = 0.0003) (Fig. 5). Univariate analyses of clinical (stage, tumor size and nodal involvement) and proteomics-based variables (6 protein signature and focal adhesion node activity) showed that focal adhesion node activity (p-value = 0.013), 6 proteins signature (p-value = 0.002) and tumor size (p-value = 0.033) have prognostic value in MIUC. Multivariate analyses showed that both focal adhesion (p-value = 0.011) and 6 proteins signature (p-value = 0.020) has independent prognostic value (Table 2).

**Table 2:** Multivariate analysis.

**Multivariate analysis**				
	Sig.	Exp(B)	95.0% IC para Exp(B)	
			Inferior	Superior
6prots	0.020	3.486	1.217	9.981
Adhesion Node	0.011	3.029	1.287	7.130
Stage	0.840	1.086	0.489	2.412
Size	0.452	0.910	0.711	1.164
N	0.747	1.150	0.492	2.687

## Discussion

The principal aim of the study was to establish a molecular classification and survival prediction in MIUC based on proteome analysis since bladder cancer classifications have generally been based on histology features^14^ and genomics have yet to be implemented in the clinic. In the clinical practice, there seems to be different groups of patients beyond pathological characteristics. Some patients, even with positive lymph nodes, may never relapse after surgery. However, other subset of patients with apparent favorable features may become metastatic. Therefore it is clear that stratification using the current system is inadequate to satisfactorily differentiate prognosis. Consequently, it is necessary to characterize MIUC patients in accordance to prognostic evolution and molecular features. The proposal of new classifications and characterization of MIUC could lead to further stratification of MIUC tumors and may drive treatment selection.

Our proteomics pipeline allowed us detecting 4,405 proteins in 58 FFPE MIUC samples. We identified groups in our protein data using sparse k-means and confirmed its consistency by random forest, supporting that different molecular subgroups exist in MIUC. Sparse k-means classification is based on 34 proteins, most of them related with focal adhesion. These two molecular groups provide additional information to clinical parameters. As we demonstrated in previous works, probabilistic graphical models using protein expression data allow characterizing differences in biological processes and pathways between groups of patients^12,15^. We were able to establish different functional nodes according to biological functions. The analysis identified 18 different functional nodes, 12 of them monitoring eleven biological processes showed differential activity between the prognostic groups previously established. These results confirm that this approach is valid to study the differential activity of biological functions between tumor groups^12^.

Group 1 showed higher expression of some proteins related with focal adhesion and extracellular matrix. Specifically, some of these proteins have been related with epithelial-to-mesenchymal transition (EMT) markers such as EH domain containing 2 (EHD2), which can inhibit metastasis by regulating cadherins^16^ or tensin 1 (TNS1), involved in focal adhesion. Low levels of TNS1 have been associated with worsening-free survival in non-muscle invasive bladder cancer^17^. Additionally, filamin A, a downstream effector of mTORC2, plays an important role in motility and invasion^18^. Additionally, we showed an increased activity of biological processes in Group 1, such as Cytoskeleton and Focal Adhesion, Metabolism and tRNA and ribosomes. It is noteworthy that related nodes, such as Cytoskeleton and Focal Adhesion nodes, and also tRNA and ribosomes nodes showed a similar behaviour, showing consistency for obtained biological information. Metabolism A node includes proteins related with negative regulation of protein metabolic process whereas Metabolism B node included proteins related to glycolysis and pyruvate metabolism, involved in generation of precursor metabolites and energy. All together, these results suggest that Group 1 have lower metastatic potential and specific features regarding metabolism and protein synthesis when compared with Group 2.

On the other hand, several proteins showed higher expression in group 2. Some immune proteins such as HSBP1 (heat shock factor binding protein 1) were associated with a decreased immune activity which may have therapeutic implications^19^. Additionally, group 2 showed increased activity in Vesicles, Transport, Proteasome, Splicing and RNA nodes. Again, we found coherence in the biological information, as long as nodes with comparable function showed similar behavior. These results suggest differences regarding intracellular trafficking, RNA processing and Proteasome activities when comparing new defined groups.

The differences in biological functions, after proper validation, could lead to develop specific treatments in concrete groups of patients. For instance, differences in metabolism could be targeted with 2-D-deoxy-glucose or metformin^20^, which are being currently tested in clinical trials for breast cancer treatment. On the other hand, proteasome targeting drugs have demonstrated therapeutic value in multiple myeloma treatment^21^.

In this study we show that the discovery of proteins as prognostic biomarkers is feasible using FFPE samples and proteomics. Indeed, we were able to identify a six protein-signature with prognosis value independently of stage, size and lymph node status. Proteins contained in this predictor are involved in multiple processes. ANXA1, a membrane-located protein, has been related with prognosis in breast cancer^22^. BGN is a protein involved in inflammation processes^23^. Niedworok *et al*.^24^ suggested that biglycan is an endogenous inhibitor of bladder cancer cell proliferation and its high expression is associated with good prognosis. PLS3 was proposed as biomarker for breast cancer prognosis^25^. To our knowledge, no previous information about MDP1, IGFBP7 and ISLR role in cancer disease has been previously reported. The prognostic value of the 6-protein signature was validated using gene expression data from another cohort.

Probabilistic graphical models allow to compare biological functions between groups but also, to build prognostic signatures. Focal adhesion functional node activity had prognostic value and split population in low and high risk of relapse. Strikingly, prognostic information provided by a traditional protein signature was complementary to information provided by focal adhesion functional node activity signature, and also to the prognostic information provided by clinical factors, as shown in the multivariate analysis. Merging these molecular features, it is feasible to establish four different risk populations. These results confirm that functional approaches could provide additional information to traditional gene/protein-centered analyses.

Our study has some limitations. Technically, proteomics provide less information when compared with genomics, thus an improvement in number of detected proteins is still necessary. On the other hand, peptide and protein identification relies in statistical parameters. Due to this, we applied strict filters for peptides/proteins selection, in order to avoid false detections, ensuring that proteins with the highest confidence in both identification and quantification are selected for analyses. Finally, although a meta-validation has been performed, these results should be validated in additional cohorts to evaluate the 6-protein signature robustness and the functional differences between new defined molecular groups. Other limitations of this study include the relatively small sample size and there may be other bias that could affect outcomes. We believe that our findings serve as important hypothesis generating findings that can be explored in future studies.

In conclusion, our approach, combining proteomics and probabilistic graphical models allow the integration of different levels of molecular information that can improve MIUC molecular characterization. We were able to differentiate two different molecular groups from our proteomics data, with different functional features that may represent new therapeutic opportunities for bladder cancer treatment. Moreover, we defined a 6 protein-signature that can predict the outcome of MIUC patients and we identified a functional node with prognosis value in MIUC, adding prognostic information to the prognostic 6-protein signature and to clinical factors.

## Methods

### Patient’s characteristics and samples selection

Patients treated at University Hospital 12 de Octubre (Madrid, Spain) were included if they had histologically documented (TNM staging^26^, T1-T4a and any N, M0) urothelial carcinoma (including of the renal pelvis, ureter, urinary bladder, or urethra). In total, 58 patients who underwent curative surgical resection between 2006 and 2012 were selected. FFPE samples were retrieved from I + 12 Biobank (RD09/0076/00118). Samples were reviewed by a genitourinary pathologist and included if cases had at least 50% of urothelial tumor cells and were invasive in the muscularis propria. The study was approved by independent review board and Ethical Committee of Hospital Universitario 12 de Octubre. All experiments were performed in accordance with relevant guidelines and regulations. Informed consent was obtained from all participants before starting treatment.

### Liquid chromatography - mass spectrometry shotgun analysis

Proteins were extracted from FFPE samples as previously described^27^. Mass spectrometry analysis was performed on a QExactive mass spectrometer coupled to a nano EasyLC 1000 (Thermo Fisher Scientific). Solvent composition at the two channels was 0.1% formic acid for channel A and 0.1% formic acid, 99.9% acetonitrile for channel B. For each sample 2 μL of peptides were loaded on a self-made column (75 μm × 150 mm) packed with reverse-phase C18 material (ReproSil-Pur 120 C18-AQ, 1.9 μm, Dr. Maisch GmbH) and eluted at a flow rate of 300 nL/min by a gradient from 2 to 35% B in 80 min, 47% B in 4 min and 98% B in 4 min. Samples were acquired in a randomized order. The mass spectrometer was operated in data-dependent mode (DDA), acquiring a full-scan MS spectra (300–1700 m/z) at a resolution of 70000 at 200 m/z after accumulation to a target value of 3000000, followed by HCD (higher-energy collision dissociation) fragmentation on the twelve most intense signals per cycle. HCD spectra were acquired at a resolution of 35000 using normalized collision energy of 25 and a maximum injection time of 120 ms. The automatic gain control (AGC) was set to 50000 ions. Charge state screening was enabled and singly and unassigned charge states were rejected. Only precursors with intensity above 8300 were selected for MS/MS (2% underfill ratio). Precursor masses previously selected for MS/MS measurement were excluded from further selection for 30 s, and the exclusion window was set at 10 ppm. The samples were acquired using internal lock mass calibration on m/z 371.1010 and 445.1200.

### Protein identification and label free protein quantification

The acquired raw MS data were processed by MaxQuant (version 1.5.2.8), followed by protein identification using the integrated Andromeda search engine. Spectra were searched against a forward Swiss Prot-human database, concatenated to a reversed decoyed fasta database and common protein contaminants (NCBI taxonomy ID9606, release date 2014-05-06). Carbamidomethylation of cysteine was set as fixed modification, while methionine oxidation and N-terminal protein acetylation were set as variable. Enzyme specificity was set to trypsin/P allowing a minimal peptide length of 7 amino acids and a maximum of two missed-cleavages. Precursor and fragment tolerance was set to 10 ppm and 20 ppm, respectively for the initial search. The maximum false discovery rate (FDR) was set to 0.01 for peptides and 0.05 for proteins. Label free quantification was enabled and a 2 minutes window for match between runs was applied. The re-quantify option was selected. For protein abundance the intensity was used, corresponding to the sum of the precursor intensities of all identified peptides for the respective protein group.

### Sparse k-means classification

Sparse k-means was used to establish differential groups between samples. Classification consistency was tested using random forest. An analysis with the Consensus Clustering algorithm^28^, applied on the data containing the variables that were selected by the sparse K-means method^29^, has provided an optimum classification into two subtypes in previous studies^30^.

### Functional network construction

Network construction was performed using probabilistic graphical models compatible with high dimensional data using correlation as associative method as previously described^12^. In order to identify functional nodes in the networks we split them in several branches and we used Gene Ontology analysis to assign a majority function to each node. Activity measurement was calculated by the mean expression of all the proteins of each node related with the assigned node function.

### Gene-Ontology Analysis

Protein to Gene Symbol conversion was performed using Uniprot and DAVID^31^. Gene Ontology Analysis was also done in DAVID selecting “*Homo sapiens*” background and GOTERM-FAT, Biocarta, KEGG and Panther databases.

### Protein signature construction

We computed a statistical significance level for each protein based on a univariate proportional hazards model with the aim of identifying proteins whose expression were significantly related to the distant metastasis-free survival (DMFS) as described previously^13^. Leave-one-out cross-validation was used to evaluate the predictive accuracy of the profiles. The cutoff point was established *a priori* and to test the statistical significance, the p-value of the log-rank test statistic for the risk groups was evaluated using 1000 random permutations. Analyses were performed in BRB-ArrayTools v4_2_1 and R v3.2.4^32^. BRB-ArrayTools has been developed by Dr. Richard Simon and BRB-ArrayTools Development Team.

### Prognostic signature meta-validation

With the aim to verify the utility of 6 protein signature, gene expression data from a MD Anderson cohort was used^4^. All probes in dataset for each gene were retrieved. Probes with higher CV were selected when multiple probes were found for a single gene, then expression values of each gene were z-score transformed as previously described^15^. To apply protein expression based signatures to gene expression values, per-gene normalization was applied as previously described^13^.

### Statistical analyses

Statistical analyses (class comparisons contingency analyses, etc.), were performed using GraphPad Prism v6 and Cytoscape^33^. Univariate and multivariate Cox regression models were performed using IBM SPSS Statistics. All p-values where two-sided, and p < 0.05 was considered statistically significant.

## Electronic supplementary material

Supplementary Figures

Supplementary Table 1

Supplementary Table 2
