# Supplementary material for: Urothelial cancer proteomics provides both prognostic and functional information
Source: Sci Rep. 2017 Nov 17;7:15819. doi: 10.1038/s41598-017-15920-6 (PMC5694001; doi:10.1038/s41598-017-15920-6)
Supplement: Supplementary file 1 — Supplementary Figures [file 41598_2017_15920_MOESM1_ESM.pdf]

Supplementary Information

Urothelial cancer proteomics provides both prognostic and functional information

Guillermo de Velasco1*$, Lucia Trilla-Fuertes2,3*, Angelo Gamez-Pozo2,3, Maria Urbanowicz4, Gustavo Ruiz-Ares1, Juan M. Sepúlveda1, Guillermo Prado-Vazquez2, Jorge M. Arevalillo5, Andrea Zapater-Moros2, Hilario Navarro5, Rocio Lopez-Vacas2, Ray Manneh1, Irene Otero1, Felipe Villacampa6,8, Jesus M. Paramio7,8, Juan Angel Fresno Vara2,3,8*, Daniel Castellano1,8*.

1Department of Medical Oncology, University Hospital 12 de Octubre, i+12, Madrid, Spain,

2Molecular Oncology & Pathology Lab, INGEMM, Instituto de Investigación Hospital La Paz-IdiPAZ, Madrid, Spain.

3Biomedica Molecular Medicine, Madrid, Spain.

4Department of Pathology, University Hospital 12 de Octubre, Madrid, Spain

5Department of Statistics, Operational Research and Numerical Analysis, University Nacional Educacion a Distancia (UNED), Spain

6 Department of Urology, University Hospital 12 de Octubre, Madrid, Spain

7 Molecular and Cell Oncology Group, Biomedical research Institute, University Hospital 12 de Octubre, i+12, and Molecular Oncology Unit, CIEMAT, Madrid, Spain,

8CIBERONC, Spain

$Corresponding Author.

*equal contribution

Corresponding author:

Guillermo de Velasco, MD PhD

Department of Medical Oncology

University Hospital 12 de Octubre

Av Cordoba s/n, 28041, Madrid, Spain

Email: gdvelasco.gdv@gmail.com

Supplementary figure 1

Classification is based in these 34 proteins. Protein Expressions quantified by MS-were significantly different ($p<0.0001$) between the two groups (high and low risk).

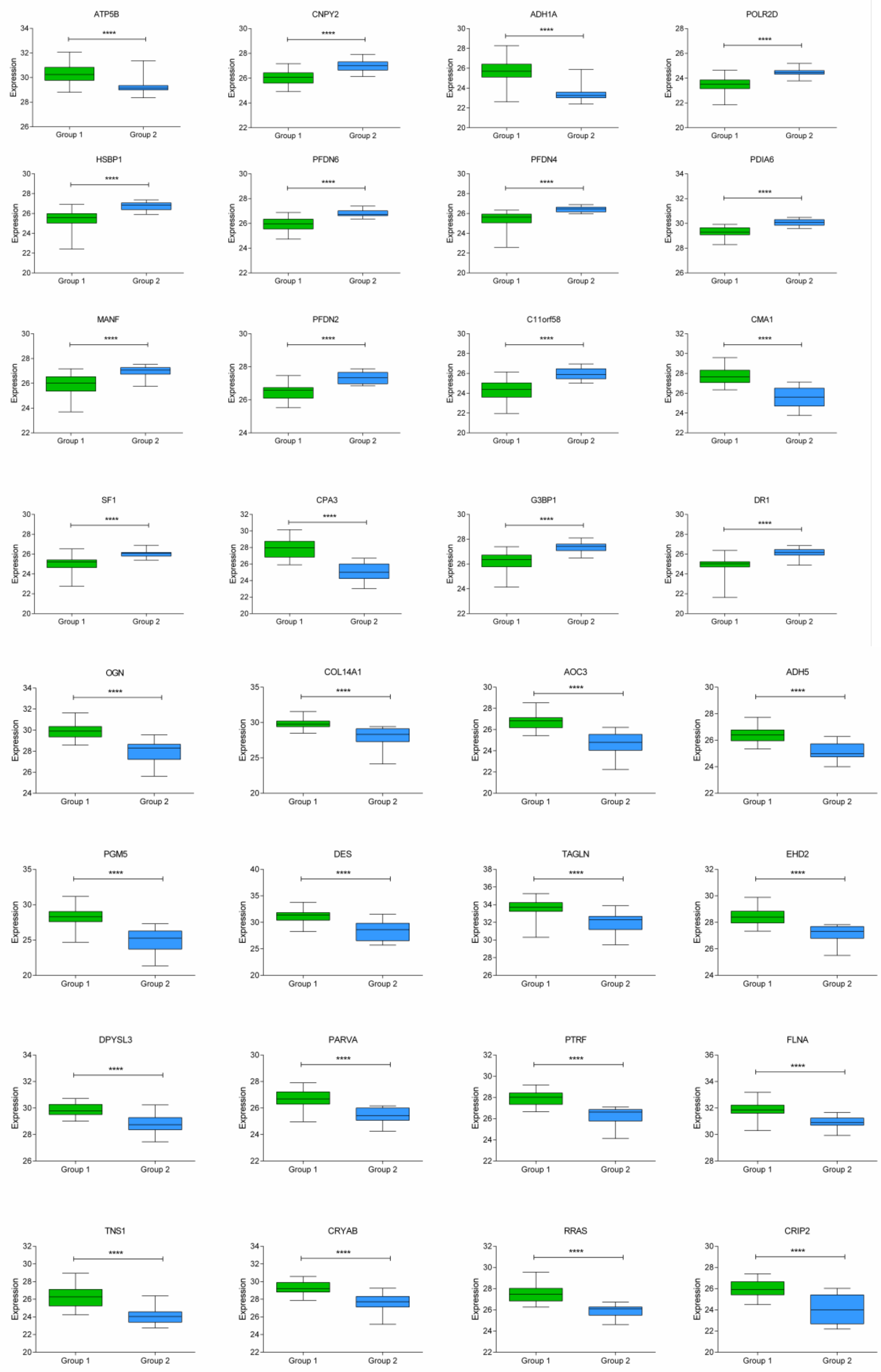
ATP5B
CNPY2
ADH1A
POLR2D
HSBP1
PFDN6
PFDN4
PDIA6
MANF
PFDN2
C11orf58
CMA1
SF1
CPA3
G3BP1
DR1
OGN
COL14A1
AOC3
ADH5
PGM5
DES
TAGLN
EHD2
DPYSL3
PARVA
PTRF
FLNA
TNS1
CRYAB
RRAS
CRIP2
Expression
Group 1
Group 2
****

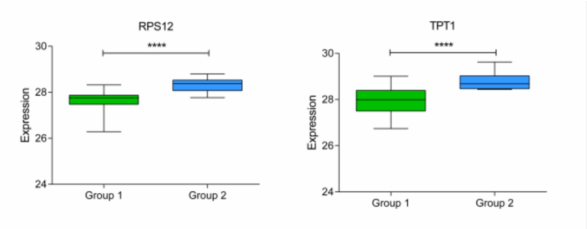

RPS12
TPT1
****
Expression
30
28
26
24
Group 1
Group 2

Supplementary figure 2

Activity measurements for each node between two groups obtained from protein data using mean expression of proteins related with the majority function. Cytoskeleton, metabolism B and A, focal adhesion, proteasome, RNA, tRNA, ribosomes, transport, vesicles and splicing nodes have different expression levels between two groups (ns $p > 0.05$, * $p \leq 0.05$, ** $p \leq 0.01$, *** $p \leq 0.001$, **** $p \leq 0.0001$).

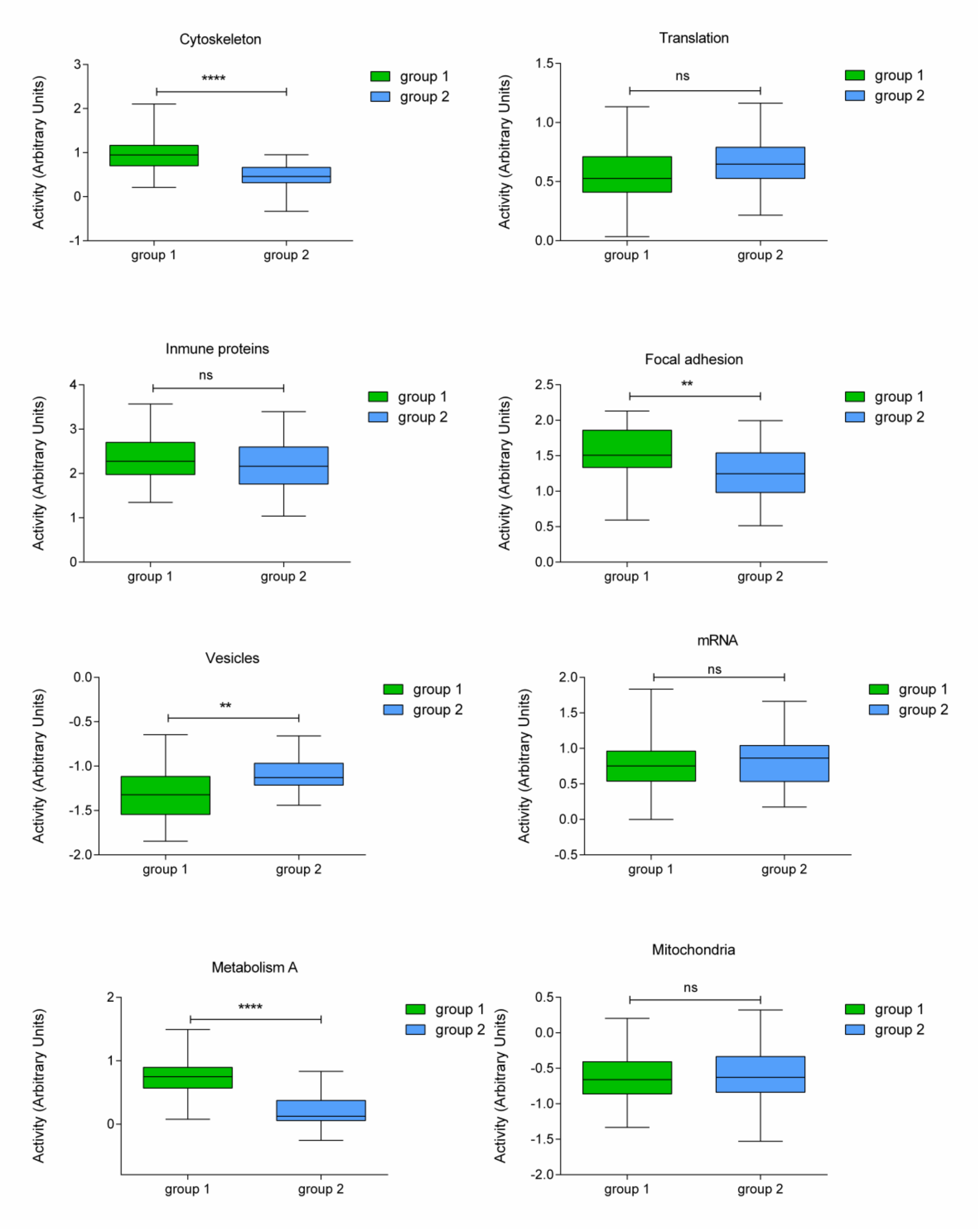

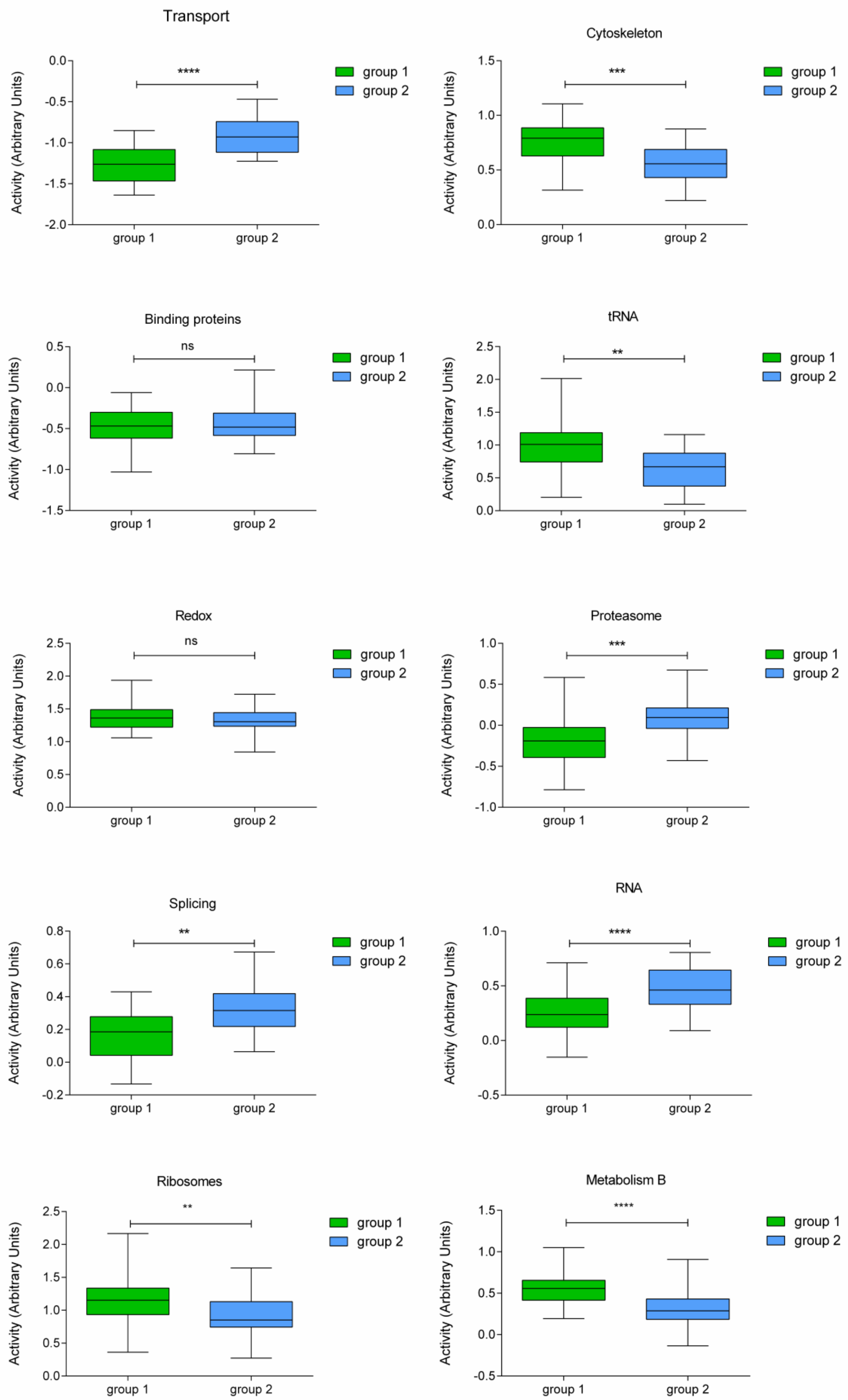

Transport
****
group 1
group 2
Activity (Arbitrary Units)
0.0
-0.5
-1.0
-1.5
-2.0
group 1
group 2
Cytoskeleton
***
group 1
group 2
Activity (Arbitrary Units)
1.5
1.0
0.5
0.0
group 1
group 2
Binding proteins
ns
group 1
group 2
Activity (Arbitrary Units)
0.5
0.0
-0.5
-1.0
-1.5
group 1
group 2
tRNA
**
group 1
group 2
Activity (Arbitrary Units)
2.5
2.0
1.5
1.0
0.5
0.0
group 1
group 2
Redox
ns
group 1
group 2
Activity (Arbitrary Units)
2.5
2.0
1.5
1.0
0.5
0.0
group 1
group 2
Proteasome
***
group 1
group 2
Activity (Arbitrary Units)
1.0
0.5
0.0
-0.5
-1.0
group 1
group 2
Splicing
**
group 1
group 2
Activity (Arbitrary Units)
0.8
0.6
0.4
0.2
0.0
-0.2
group 1
group 2
RNA
****
group 1
group 2
Activity (Arbitrary Units)
1.0
0.5
0.0
-0.5
group 1
group 2
Ribosomes
**
group 1
group 2
Activity (Arbitrary Units)
2.5
2.0
1.5
1.0
0.5
0.0
group 1
group 2
Metabolism B
****
group 1
group 2
Activity (Arbitrary Units)
1.5
1.0
0.5
0.0
-0.5
group 1
group 2

Supplementary Figure 3

6 proteins signature validation in MD Anderson cohort (HR 2.10 95% CI 0.2-1.1; [p=0.04]).

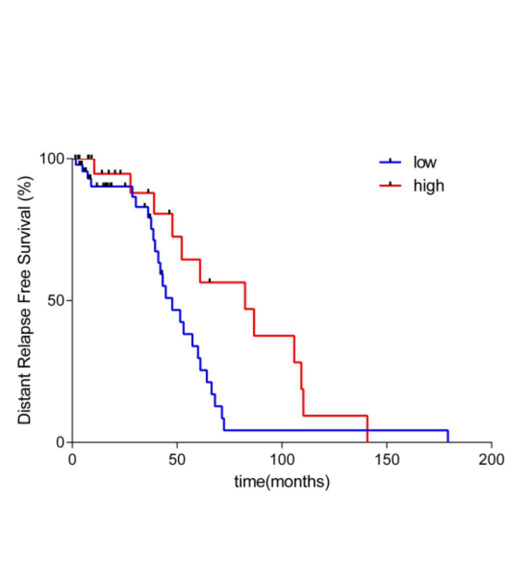

Log-rank (Mantel-Cox) test		
Chi square	4.654	
df	1	
P value	0.0310	
P value summary	*	
Are the survival curves sig different?	Yes	
Gehan-Breslow-Wilcoxon test		
Chi square	4.032	
df	1	
P value	0.0446	
P value summary	*	
Are the survival curves sig different?	Yes	
Median survival		
low	47.70	
high	82.40	
Ratio (and its reciprocal)	0.5789	1.727
95% CI of ratio	0.2921 to 1.147	0.8716 to 3.424
Hazard Ratio (Mantel-Haenszel)	A/B	B/A
Ratio (and its reciprocal)	2.100	0.4762
95% CI of ratio	1.070 to 4.120	0.2427 to 0.9344
Hazard Ratio (logrank)	A/B	B/A
